# Short-Term Gingival Microcirculatory Responses to Non-Invasive Physical Stimulation: Implications for Accelerated Orthodontic Research

**DOI:** 10.3390/dj14060353

**Published:** 2026-06-09

**Authors:** Shuichi Atsuta, So Koizumi, Shun-suke Takahashi, Satoko Wada-Takahashi, Kazuhide Seimiya, Masatoshi Shimura, Hayato Furuhashi, Manami Yamaguchi, Keiichi Tsukinoki, Masahiro Takahashi, Tetsutaro Yamaguchi

**Affiliations:** 1 Department of Orthodontics, Graduate School of Dentistry, Kanagawa Dental University, Yokosuka 238-8580, Japan; koizumi@kdu.ac.jp (S.K.); furuhasi@kdu.ac.jp (H.F.); yamaguchi.manami@kdu.ac.jp (M.Y.); takahashi.masahiro@kdu.ac.jp (M.T.); t.yamaguchi@kdu.ac.jp (T.Y.); 2 Department of Pharmacology, Kanagawa Dental University, Yokosuka 238-8580, Japan; takahashi.shunsuke@kdu.ac.jp; 3 Department of Oral Physiology, Kanagawa Dental University, Yokosuka 238-8580, Japan; s.takahashi@kdu.ac.jp; 4 Division of Dental Technology, Department of Dental Clinical Support, School of Dentistry, Kanagawa Dental University, Yokosuka 238-8580, Japan; seimiya@kdu.ac.jp (K.S.); m.shimura@kdu.ac.jp (M.S.); 5 Department of Environmental Pathology, Kanagawa Dental University, Yokosuka 238-8580, Japan; tsukinoki@kdu.ac.jp

**Keywords:** orthodontics, accelerated orthodontics, tooth movement, gingival blood flow, microcirculation, physical stimulation, vibration, laser Doppler flowmetry

## Abstract

**Background/Objectives**: Acceleration of orthodontic tooth movement remains a major challenge in clinical orthodontics. Evidence suggests that increased local blood flow around the alveolar bone is key to bone remodeling and potentially reflects early biological responses associated with accelerated orthodontics. This study aimed to investigate the effects of non-invasive physical stimuli on gingival microcirculation. **Methods**: Eight healthy adult male volunteers were included in the analysis. Gingival blood flow was assessed using laser Doppler flowmetry under the following conditions: no-stimulation condition (None) and four types of stimuli: thermal stimulation (THM), electric field stimulation (ELF), vibration stimulation (VIB), and far-infrared stimulation (FIR). Gingival blood flow was recorded before and after each stimulation, and the rate of change was calculated. Statistical analysis was performed using a linear mixed-effects model with Type III ANOVA (Satterthwaite approximation), followed by Dunnett-adjusted comparisons. **Results**: A statistically significant difference was observed between stimulation conditions (*p* = 0.0087). VIB significantly increased gingival blood flow compared with the no-stimulation condition (*p* = 0.0041), whereas ELF showed a trend toward increased blood flow (*p* = 0.0936); THM and FIR showed no statistically significant effects. **Conclusions**: The findings of this study suggest that non-invasive physical stimuli, particularly vibration stimulation, can enhance gingival microcirculation. Although tooth movement was not directly evaluated, the observed hemodynamic changes may represent short-term physiological responses to non-invasive physical stimulation.

## 1. Introduction

The number of patients undergoing orthodontic treatment has increased in recent years. However, because orthodontic tooth movement depends on alveolar bone remodeling, treatment duration typically ranges from 2 to 3 years. As a result, prolonged orthodontic treatment increases patient burden and is associated with increased risks, such as dental caries and periodontal disease [1,2]. Therefore, accelerating tooth movement and shortening treatment duration have become important research objectives in orthodontics. These approaches are collectively referred to as “accelerated orthodontics.”

Accelerated orthodontics stems from the clinical observation that alveolar bone surgery enhances tooth movement. In 1959, Köle reported that corticotomy of the alveolar cortical bone enabled en bloc movement of teeth with the surrounding bone, thereby accelerating tooth movement [3]. Subsequently, Periodontally Accelerated Osteogenic Orthodontics (PAOO), proposed by Wilcko et al., stimulates alveolar bone metabolism by combining corticotomy with bone grafting [4]. Although effective in reducing treatment time, these surgical approaches are highly invasive, associated with increased patient burden, and have limited indications.

Regional acceleration phenomenon (RAP) is a key biological mechanism underlying accelerated orthodontics. Frost has proposed RAP as a transient increase in local bone metabolism following a surgical insult, characterized by enhanced osteoclastic and osteoblastic activity and a temporary bone density reduction [5]. This process is accompanied by increased inflammatory cytokine expression, vasodilation, and enhanced local blood flow [6,7,8]. Increased blood flow facilitates the delivery of oxygen and nutrients and promotes the recruitment and activation of bone-related cells, thereby contributing to tooth movement [9,10,11]. Accordingly, blood flow changes may serve as an indicator of early responses in accelerated orthodontics.

In addition to conventional surgical techniques, less invasive methods, such as piezocision and micro-osteoperforation (MOP), have been proposed [12]. These approaches aim to induce RAP with minimal injury to the alveolar cortical bone; however, their reproducibility and long-term safety remain controversial. More recently, nonsurgical approaches employing physical stimuli, such as low-amplitude vibration stimulation, low-level laser therapy, electrical stimulation, and ultrasound, have attracted attention [13,14,15,16,17]. Although these modalities may influence bone metabolism and cellular activity, early-stage microcirculatory responses remain poorly understood.

Gingival blood flow has traditionally been evaluated using indirect and invasive methods [18]. Recently, laser Doppler flowmetry (LDF) has been widely adopted as a non-invasive, real-time technique to assess microcirculation [18,19]. LDF measures microvascular blood flow using laser light scattering and Doppler shifts from red blood cells, enabling sensitive detection of changes in local perfusion [20,21].

As a non-invasive, painless method, LDF enables reliable evaluation of blood flow in oral tissues, including the gingiva and dental pulp, without disrupting microcirculation [21,22,23]. Recent systematic reviews have identified LDF as a representative and reliable method for assessing oral soft tissue blood flow [24].

In this study, we focused on multiple physical stimuli that could increase local blood flow using non-invasive methods. Four types of stimulation—thermal, electric field, vibration, and far-infrared radiation—were applied to the maxillofacial region, and changes in gingival blood flow were evaluated. These stimuli are nonsurgical and non-invasive, and can be repeatedly applied, suggesting potential applicability in clinical settings.

The aim of this study was to evaluate the effects of four types of non-invasive physical stimulation on gingival microcirculation and determine the potential use of changes in blood flow as an indicator of early physiological responses in accelerated orthodontics. Although tooth movement was not directly assessed, this study focused on hemodynamic changes preceding bone remodeling to provide novel insights into the mechanistic basis of non-invasive accelerated orthodontics.

## 2. Materials and Methods

### 2.1. Participants

This study was conducted in accordance with the Declaration of Helsinki and approved by the Ethics Committee of Kanagawa Dental University (Approval No. 1161). Written informed consent was obtained from all participants after a full explanation of the study was provided.

Eight healthy adult males were enrolled in this study. This study was designed as an exploratory pilot study to estimate effect sizes and assess the feasibility of future research; therefore, no a priori sample size calculations were performed. Before participation, a questionnaire was administered to assess the presence of systemic diseases, regular medication use, and smoking history. Because periodontal inflammation may affect gingival microcirculation, only adult volunteers with clinically healthy gingival tissues were included. Furthermore, to minimize the influence of aging on blood flow, participants were restricted to those aged 28–31 years (mean age: 28.7 years).

The exclusion criteria were as follows: occlusal trauma, suspected bruxism, significant defects or premature contact with the target or opposing teeth, prominent alveolar exostosis, gingival discoloration or morphological abnormalities, jaw deformities, temporomandibular disorders, missing teeth other than the third molars, and history of smoking within the past 6 months. Additionally, female participants were excluded because fluctuations in estrogen levels associated with the menstrual cycle influence vascular reactivity [25].

### 2.2. Measurement of Gingival Blood Flow

**Figure 1:**
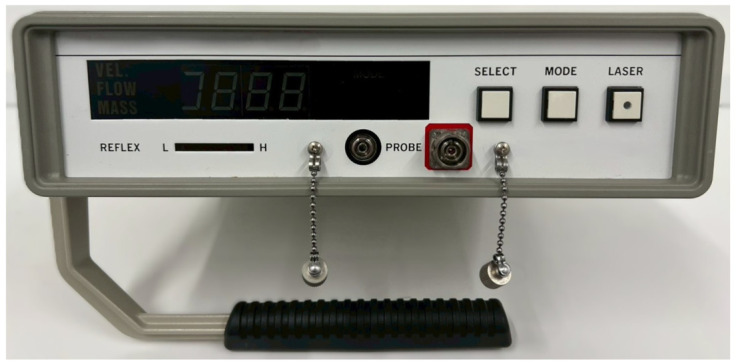
Laser Doppler flowmeter used for measuring gingival blood flow.

**Figure 2:**
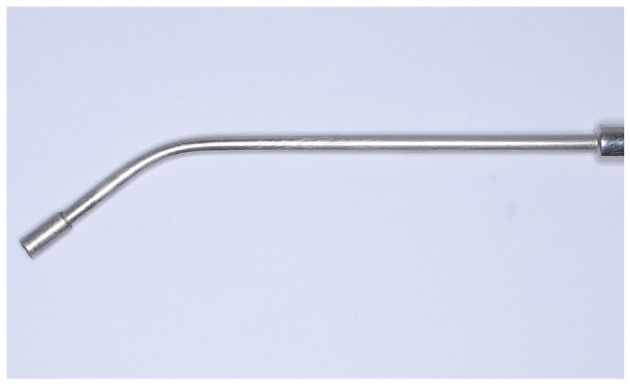
Non-contact probe used for gingival blood flow measurement.

Gingival blood flow (mL/min/100 g) was measured using a laser Doppler flowmeter (Omegaflo Flo-N1; Omega Wave Co., Ltd., Tokyo, Japan) with a non-contact probe (Figure 1 and Figure 2).

**Figure 3:**
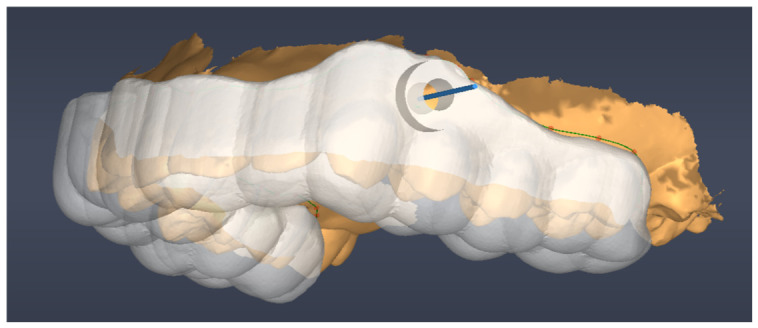
Splint design using DentiqGuide.

To ensure measurements at the same site before and after stimulation, a custom-made splint was fabricated to fix the probe position. Optical impressions of the maxilla were obtained with an intraoral scanner (iTero Element 2; Align Technology, Inc., San Jose, CA, USA), and the resulting STL data were used to design a splint with DentiqGuide (Ci Digital Solutions Co., Ltd., Tokyo, Japan) (Figure 3).

The measurement site was defined as the buccally attached gingiva located at the midpoint between (1) a point 3 mm apical to the gingival margin of the maxillary left canine and (2) a point 3 mm apical to the gingival margin of the maxillary left first premolar.

**Figure 4:**
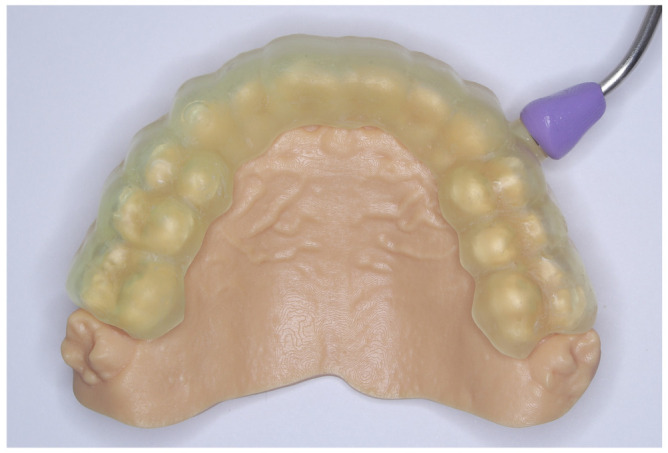
Splint for probe fixation.

The splint was fabricated using a 3D printer (S-WAVE IMD-S; Shofu Inc., Kyoto, Japan) with biocompatible resin material (S-WAVE Print Surgical Guide HT; Shofu Inc., Kyoto, Japan) based on STL data obtained from intraoral scanning. The splint was designed to fit the buccal aspect of the maxillary dentition and oral vestibule and included a guide hole for probe placement. To ensure reproducible positioning of the splint during repeated measurements, silicone bite registration material (Delikit, Sherpa Korea Co., Ltd., Seoul, Republic of Korea) was applied to the occlusal surfaces of the opposing mandibular teeth to create individualized occlusal indentations. This allowed the participant to reproduce the same occlusal position at each measurement session. The laser Doppler flowmetry probe was inserted into the guide hole and stabilized with silicone material to maintain a constant position, angle, and distance relative to the gingival surface throughout the measurements (Figure 4).

**Figure 5:**
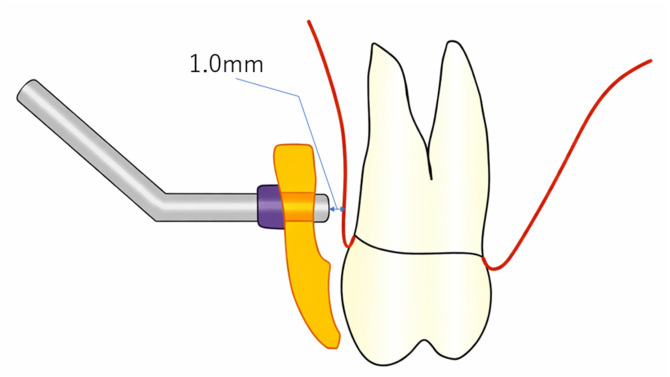
Method for probe fixation.

The probe was positioned perpendicular to the gingival surface and placed 1.0 mm from the gingival mucosa (Figure 4 and Figure 5).

### 2.3. Experimental Procedure

All measurements were performed in a quiet room under controlled environmental conditions (temperature: 20–25 °C; relative humidity: 50–70%). The participants were placed supine on a dental chair, with their heads adjusted to the same level as the heart. To prevent contact between the lips and the probe, the lips were retracted using silicone material. Participants were instructed to refrain from eating, drinking, and brushing their teeth for at least 1 h before measurements. This restriction was introduced to minimize transient changes in the gingival microcirculation induced by oral mechanical or thermal stimulation. Previous laser Doppler flowmetry studies have shown that tooth brushing and other mechanical or thermal stimuli can alter gingival blood flow [26,27,28]. Although no universally established restriction period exists for gingival blood flow measurements, a 1 h interval was selected as a conservative period to standardize baseline conditions across participants and measurement sessions. To minimize the influence of circadian variation, all measurements were performed between 17:00 and 20:00. Peripheral and oral microcirculation are known to exhibit physiological fluctuations associated with circadian rhythm, autonomic nervous system activity, vascular tone regulation, and body temperature changes. Therefore, standardizing the measurement time was considered important to reduce physiological variability in gingival blood-flow measurements [29].

After wearing the splint for probe fixation, participants rested for at least 10 min. Baseline gingival blood flow was recorded for 30 s, and the mean value was used as the baseline. Subsequently, the following stimulation conditions were applied: thermal stimulation (THM), electric field stimulation (ELF), vibration stimulation (VIB), far-infrared stimulation (FIR), and a no-stimulation condition (None).

THM: Azukinochikara (Kobayashi Pharmaceutical Co., Ltd., Osaka, Japan);
ELF: DENBA Health (DENBA JAPAN Co., Ltd., Tokyo, Japan);
VIB: SureSmile^®^ VPro™ (Dentsply Sirona Inc., Charlotte, NC, USA);
FIR: BAKUNE (TENTIAL Co., Ltd., Tokyo, Japan).

For thermal stimulation (THM), the product was heated in a microwave for 30 s and applied for 15 min to cover the entire face.

For electric field stimulation (ELF), the participants lay supine on a dedicated mat, and the device was activated to apply whole-body electric field stimulation for 15 min.

For vibration stimulation (VIB), the vibration device was placed between the dental arches while the splint was in place, providing continuous stimulation for 15 min.

For far-infrared stimulation (FIR), the participants wore specialized clothing and remained supine for 15 min.

Gingival blood flow was measured for 30 s, starting 14 min following the onset of stimulation, and the mean value was defined as the post-stimulation value. The measurement duration (30 s) was selected based on previous studies demonstrating that stable LDF signals can be obtained within this timeframe [23]. Using the mean value over 30 s reduced signal variability. The same protocol was performed under the no-stimulation condition to evaluate time-dependent changes in blood flow. To minimize the influence of participant awareness, the timing of blood flow measurements (before and after stimulation) was not disclosed, and participants were blinded to it.

The order of the stimulation conditions was randomized to eliminate order effects. To avoid carryover effects, each stimulation condition was tested on a separate day with an interval of at least 24 h between sessions because stimulation-induced gingival blood-flow responses have been reported to be transient and recover toward baseline within a relatively short period after stimulation [26,27,28]. All measurements were performed by a single examiner, and probe positioning was standardized using a custom splint to ensure reproducibility. Experimental conditions (posture, environment, and measurement time) were standardized to minimize external influences.

### 2.4. Statistical Analysis

Statistical analyses were performed using R software version 4.5.2 (R Foundation for Statistical Computing, Vienna, Austria). The response for each stimulation condition was calculated as the percentage change from the baseline:

[(Post − Baseline)/Baseline] × 100

A linear mixed-effects model was constructed, with subjects treated as random effects and stimulation conditions used as fixed effects. The significance of the fixed effects was evaluated using Type III analysis of variance (ANOVA) with Satterthwaite approximation. Multiple comparisons were performed using Dunnett’s test, with the no-stimulation condition as the control, based on the estimated marginal means. The model validity was assessed by examining the normality and homoscedasticity of the residuals. The significance level was set at 5%.

## 3. Results

First, to examine the effects of different stimulation conditions on the rate of change (ChangeRate), a linear mixed-effects model was constructed with subjects as a random effect and stimulation conditions (Stim) as a fixed effect. The results of Type III analysis of variance (ANOVA) showed a statistically significant main effect of the stimulation condition (F(4, 35) = 4.02, *p* = 0.0087) (Table 1).

Next, to verify the model’s assumptions, the normality of the residuals was assessed using the Shapiro–Wilk test. The results indicated that the residuals were normally distributed (W = 0.97, *p* = 0.32), suggesting that the model was appropriate.

**Figure 6:**
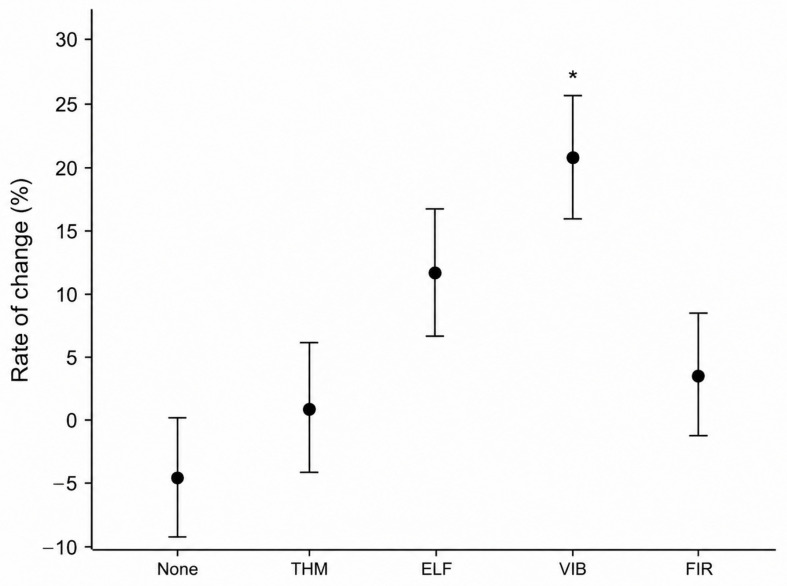
Estimated marginal means of the rate of change for each stimulation condition. Data are presented as mean ± standard error (mean ± SE). * *p* < 0.05 compared with the no-stimulation condition (Dunnett’s test).

Estimated marginal means for each stimulation condition were calculated using a linear mixed-effects model. Compared with the no-stimulation condition, ELF and VIB showed higher rates of change, with VIB showing the greatest increase among all conditions. In contrast, THM and FIR exhibited relatively small increases (Figure 6).

**Figure 7:**
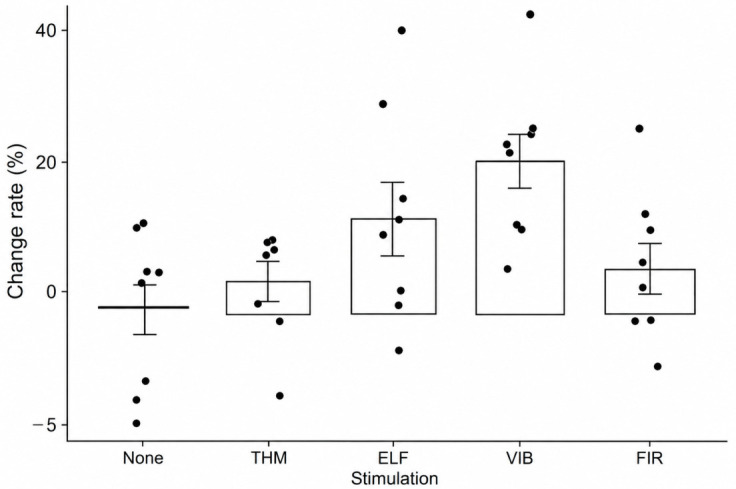
Individual changes in gingival blood flow (%) under each stimulation condition in individual subjects.

Furthermore, variability among subjects was observed in the rate of change under each stimulation condition (Figure 7).

Additionally, multiple comparisons were performed using Dunnett’s test based on the estimated marginal means from the linear mixed-effects model, with the no-stimulation condition as the reference. The results showed that VIB had a significantly higher rate of change than the no-stimulation condition (difference = 20.67%, *p* = 0.0041). In contrast, ELF showed an increasing trend, although it did not reach statistical significance (difference = 13.14%, *p* = 0.0936). No statistically significant differences were observed for THM or FIR compared with the no-stimulation condition (Table 2).

Overall, the stimulation conditions had a statistically significant effect on the rate of change in gingival blood flow. Among the tested conditions, vibration stimulation (VIB) showed the largest increase and was the only condition that demonstrated a statistically significant difference compared with the control condition.

## 4. Discussion

In this study, we investigated the effects of various non-invasive stimuli on the rate of change in gingival blood flow. The results demonstrated a statistically significant difference between stimulation conditions, with VIB showing the largest increase in gingival blood flow and a statistically significant difference compared with the no-stimulation condition. The effect size for VIB was large (Cohen’s d = 0.92). In contrast, ELF showed a trend toward increased effects but did not reach statistical significance, whereas THM and FIR showed no clear effects. The statistically significant increase in blood flow observed with the VIB may be attributed to the promotion of vasodilatory responses induced by mechanical stimulation. Vibration has been reported to act on vascular endothelial cells, thereby enhancing nitric oxide (NO) production [14,30], leading to relaxation of vascular smooth muscles and increased local blood flow.

In addition, vibration stimulation may stimulate mechanoreceptors and cells within the periodontal ligament, thereby activating local circulation and bone metabolism [31]. The present findings are consistent with these reports and support the potential of vibration stimulation to enhance microcirculation in periodontal tissues.

In contrast, ELF showed a relatively large increase in blood flow but did not reach statistical significance. This may be attributed to the limited statistical power owing to the small sample size and inter-individual variability. Indeed, variability among the subjects was observed across all stimulation conditions in this study, suggesting that the statistical power might have been insufficient to detect statistically significant differences. Nevertheless, the mean values showed an increasing trend relative to the no-stimulation condition, suggesting that ELF may exert a physiological effect on blood flow, although further investigation is warranted.

No clear changes were observed in the THM or FIR. Although thermal stimulation and far-infrared radiation increase blood flow through vasodilation [17,32], the intensity or duration of stimulation in the present study might have been insufficient to induce measurable effects. Furthermore, the gingival tissue has a different regulatory mechanism of blood flow than the skin; therefore, its responsiveness to the same stimuli may differ. Each stimulus used in this study promoted blood flow via a different mechanism. Thermal stimulation increases blood flow by inducing vasodilation via thermoregulatory mechanisms, improving microcirculation [17]. Electric field stimulation alters the dipole orientation of water molecules and induces water rearrangement at the cell membrane interface, potentially influencing cellular functions and the local environment [33]. Vibration stimulation promotes blood flow by activating cells and mechanoreceptors within periodontal tissues, thereby enhancing local circulation and bone metabolism [31]. Far-infrared radiation has been reported to promote blood flow primarily through thermal effects, including increased skin temperature and vasodilation [32].

Among these stimuli, vibration demonstrated the most pronounced effect on gingival microcirculation in this study. However, direct comparisons among stimuli should be interpreted with caution because their mechanisms of action, stimulation sites, and delivery methods differed. In the present study, each stimulation modality was applied according to its typical clinical or commercially intended usage condition. Vibration stimulation was locally applied to the dental arches because orthodontic vibration devices are generally designed to stimulate the dentition and periodontal tissues directly. In contrast, ELF and FIR devices are commonly intended for broader regional or whole-body applications, whereas THM was applied locally to the facial region.

Therefore, the observed differences among conditions may reflect not only differences in biological effects but also differences in stimulation site and application method. VIB stimulation might have acted more directly at the measurement site, whereas the other stimuli might have exerted indirect or systemic effects. This may be attributable to the fact that the vibration device used in the present study was originally designed to deliver direct stimulation to the teeth for the purpose of accelerating orthodontic tooth movement. To establish conditions more comparable to ELF and FIR stimulation, the use of a whole-body vibration device might have allowed greater standardization of the stimulation conditions. Future studies using more standardized stimulation sites and application conditions may provide further insight into the differences among stimulation modalities. However, the primary aim of this exploratory study was not to establish strict equivalence among stimulation sites, but rather to evaluate gingival microcirculatory responses under practical application conditions for each modality. Accordingly, the present findings should not be interpreted as demonstrating the superiority of one stimulus over another, but rather as reflecting differences in short-term hemodynamic responses under the present experimental conditions. These findings suggest that external stimuli can modulate blood flow in periodontal tissues and may enhance local blood flow associated with bone remodeling during orthodontic treatment.

Recent evidence suggests that high-frequency vibration may enhance orthodontic tooth movement by stimulating cellular activity and bone remodeling processes; however, the clinical effectiveness of vibration-assisted orthodontics remains controversial [15,34]. The present findings partially agree with studies reporting the biological effects of vibration stimulation, as VIB produced the largest increase in gingival blood flow among all stimulation conditions and was the only modality that demonstrated a statistically significant difference compared with the no-stimulation condition. Increased local blood flow may support early physiological responses associated with tissue remodeling by promoting oxygen and nutrient delivery and enhancing local cellular activity. However, our findings do not directly support the clinical effectiveness of vibration-assisted orthodontics in accelerating tooth movement because actual tooth movement and bone metabolic activity were not evaluated in this study. The discrepancy between biological responses and clinical outcomes reported in previous studies may be related to differences in vibration frequency, stimulation duration, application protocols, observation periods, and outcome measures. Furthermore, gingival blood-flow responses may represent only an early microcirculatory reaction and may not necessarily correlate with clinically measurable acceleration of orthodontic tooth movement. Therefore, although the present results support the biological plausibility of vibration-induced tissue responses, the relationship between microcirculatory changes and clinically significant orthodontic acceleration remains unclear.

Previous studies have suggested a relationship between increased blood flow and accelerated tooth movement. Zheng et al. reported that low-level laser therapy (LLLT) increased orthodontic tooth movement by approximately 34%, potentially through enhanced mitochondrial activity, increased ATP production, and vasodilation induced by nitric oxide (NO) release [13]. In addition, Asagai et al. demonstrated that LLLT increased blood flow by approximately 8.7–23.5% [35]. This study was an exploratory investigation focusing on short-term changes in gingival blood flow and did not directly evaluate tooth movement, bone metabolic markers, or inflammatory molecules. Therefore, the clinical significance of the observed changes in blood flow should be interpreted with caution.

Nevertheless, this study provides important insights into early hemodynamic responses to non-invasive physical stimulation. The observed changes in blood flow may reflect early physiological responses associated with tissue remodeling and suggest that gingival blood-flow changes may serve as an indicator of early physiological responses. Future studies with larger sample sizes, longitudinal designs, and direct evaluation of tooth movement and bone metabolism are needed to address these limitations and further clarify the relationship between blood flow changes and accelerated orthodontic responses.

In recent years, artificial intelligence (AI) has emerged as one of the most extensively studied digital technologies in the field of orthodontics [36]. However, because the present study was designed as an exploratory pilot study with a relatively small sample size, conventional statistical methods were considered more appropriate than AI-based analytical approaches. In general, machine-learning models applied to limited datasets are prone to overfitting and may result in reduced interpretability of the findings. Future studies with larger datasets may benefit from the application of AI and machine-learning techniques to identify complex patterns in gingival microcirculatory responses and predict individual variability in response to physical stimulation. At the same time, careful consideration should be given to potential limitations, including overfitting, reduced interpretability, and excessive reliance on automated analyses. Therefore, AI-generated findings should not be accepted uncritically but should instead be validated through appropriate scientific and clinical evaluation to ensure their reliability and relevance.

This study has several limitations. First, the sample size was small because this was designed as an exploratory pilot study, which may have limited the statistical power and generalizability of the findings. In addition, the evaluation focused on short-term changes in gingival blood flow, and the persistence and temporal dynamics of these responses were not assessed. Molecular biological markers related to inflammation and bone metabolism were also not evaluated. Furthermore, the stimulation conditions were not fully equivalent across modalities, which may have influenced the observed differences in gingival blood-flow responses. VIB was directly applied to the dentition and periodontal tissues, whereas ELF and FIR were applied more systemically or regionally.

In addition, the study population consisted only of healthy young adult males, which may limit the applicability of the findings to broader populations. Female participants were excluded to minimize the influence of hormonal fluctuations on vascular responses. However, because a large proportion of orthodontic patients are female [37], future studies should investigate gingival blood-flow responses in women under various physiological conditions, including different phases of the menstrual cycle, pregnancy, and menopause. Future studies with larger and more diverse cohorts, longitudinal designs, and comprehensive biological assessments are needed to further clarify the physiological significance of these microcirculatory changes.

## 5. Conclusions

In this study, we investigated the effects of non-invasive physical stimulation on changes in gingival blood flow and observed a significant effect of the stimulation condition on gingival microcirculation, with VIB showing the greatest increase in gingival blood flow and a statistically significant difference compared with the no-stimulation condition. These findings indicate that non-invasive physical stimuli can influence microcirculation in periodontal tissues. Although this study did not directly evaluate tooth movement, the observed changes in gingival blood flow may represent short-term physiological responses to non-invasive physical stimulation.

## Figures and Tables

**Table 1:** Analysis of variance results for the rate of change among stimulation conditions.

Factor	Degrees of Freedom	F Value	*p* Value
Stim	4, 35	4.02	0.0087

**Table 2:** Comparisons between each stimulation condition and the no-stimulation condition (Dunnett’s test).

Comparison	Difference (%)	*p* Value
THM–None	4.32	0.8638
ELF–None	13.14	0.0936
VIB–None	20.67	0.0041 *
FIR–None	6.38	0.6346

* *p* < 0.05 compared with the no-stimulation condition.

## Data Availability

The data presented in this study are available from the corresponding author upon request.
